# Supplementary material for: Voltage controlled Néel vector rotation in zero magnetic field
Source: Nat Commun. 2021 Mar 15;12:1674. doi: 10.1038/s41467-021-21872-3 (PMC7960997; doi:10.1038/s41467-021-21872-3)
Supplement: Supplementary file 1 — Supplementary Information [file 41467_2021_21872_MOESM1_ESM.pdf]

Supplementary Information

Voltage controlled Néel vector rotation in zero magnetic field

Ather Mahmood[1], Will Echtenkamp[1], Mike Street[1], Jun-Lei Wang[1], Shi Cao[1], Takashi Komesu[1], Peter A. Dowben[1], Pratyush Buragohain[1], Haidong Lu[1], Alexei Gruverman[1], Arun Parthasarathy[2], Shaloo Rakheja[3], Christian Binek[1,*]

[1]*Department of Physics & Astronomy and the Nebraska Center for Materials and Nanoscience, University of Nebraska-Lincoln, Lincoln, NE 68588-0299, USA*

[2]*Department of Electrical Engineering, New York University, Brooklyn, New York 11201, USA*

[3]*Holonyak Micro and Nanotechnology Laboratory, University of Illinois at Urbana–Champaign, Urbana, IL 61801, USA*

*To whom correspondence should be addressed. E-mail: cbinek@unl.edu

Supplementary Note 1. Magneto-Optical Kerr Measurements (MOKE)

Fig. S1 shows the *T*-dependence of the coercive field, $\mu_0 H_c$, of a CoPd ferromagnetic (FM) constituent film which is part of a $Cr_2O_{3-x}B_x$ (100nm)/CoPd heterostructure with $x \approx 0.1$. The FM hysteresis loops of CoPd have been measured at various temperatures using polar magneto-optical Kerr effect. Below the blocking temperature of T_B= 355 K, loop broadening sets in. It is associated with exchange coupling between the interface magnetizations of the AFM and FM films indicating that AFM order has been established for $T \leq 355$ K [1]. This implies $T_N(Cr_2O_{3-x}B_x) \geq 355$ K > $T_N(Cr_2O_3) = 307$ K. However, despite the presence of exchange shift of the perpendicular CoPd hysteresis loop along the magnetic field axis is absent. Presence of exchange coupling in the absence of a loop shift by an EB field, $\mu_0 H_{EB}$, is repeatedly observed for FM films with perpendicular anisotropy.

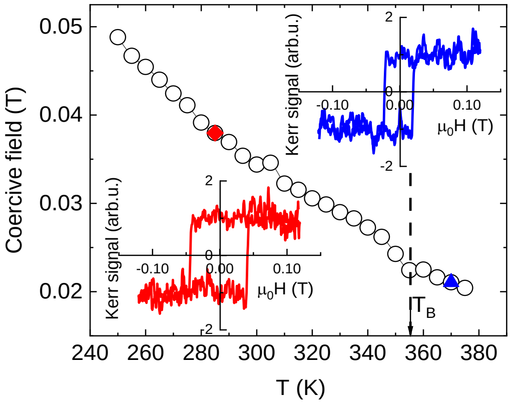

Fig. S1: **Magnetometry of B: Cr_2O_3/CoPd perpendicular exchange bias heterostructure**. $\mu_0 H_c$, versus *T* of hysteresis loops of CoPd film in proximity of $Cr_2O_{3-x}B_x$ (100nm). Blocking temperature T_B=355 K is indicated by an arrow and a vertical dashed line. Insets show representative loop at 285 K > T_B (lower inset and red diamond data point in H_c vs *T*) and 370 K < T_B (upper inset and blue triangle data point in H_c vs *T*) measured by polar Kerr effect, respectively.

Supplementary Note 2. Inverse Photoemission data

Fig. S2 shows the spin integrated spectra for single crystal chromia and B-doped chromia thin films. While bulk chromia is an exceedingly good dielectric [2], the B:Cr_2O_3 film (Figure S2, black line) exhibits significant broadening of the intensity versus energy. This broadening of the spectral intensity is indicative of surface charging, leading to a decrease of the effective incident electron kinetic energy. This is characteristic of increased sample resistivity. The inverse photoemission spectra from the undoped Cr_2O_3 single crystal (Figure S2, blue line), is very similar to the spectra taken for undoped Cr_2O_3 thin films [3], but is here seen to be characteristic of an n-type material (electron doped), a signature of oxygen vacancies.

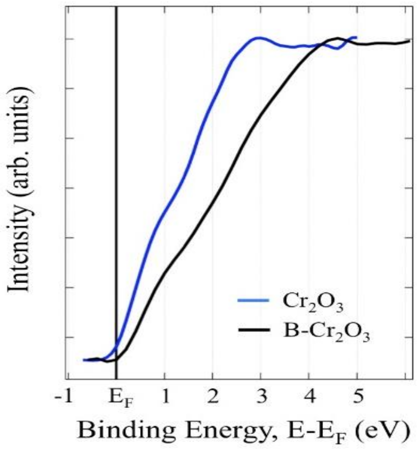

Fig. S2: **Inverse photoemission data for films of pure and B-doped chromia.** The B:Cr_2O_3 film (black line) exhibits significant broadening of the intensity vs. energy profile when compared with the data of undoped Cr_2O_3 (blue line). The broadening originated from charging of the sample which is indicative of increased resistivity.

Supplementary Note 3. Transport measurements on Hall bar devices

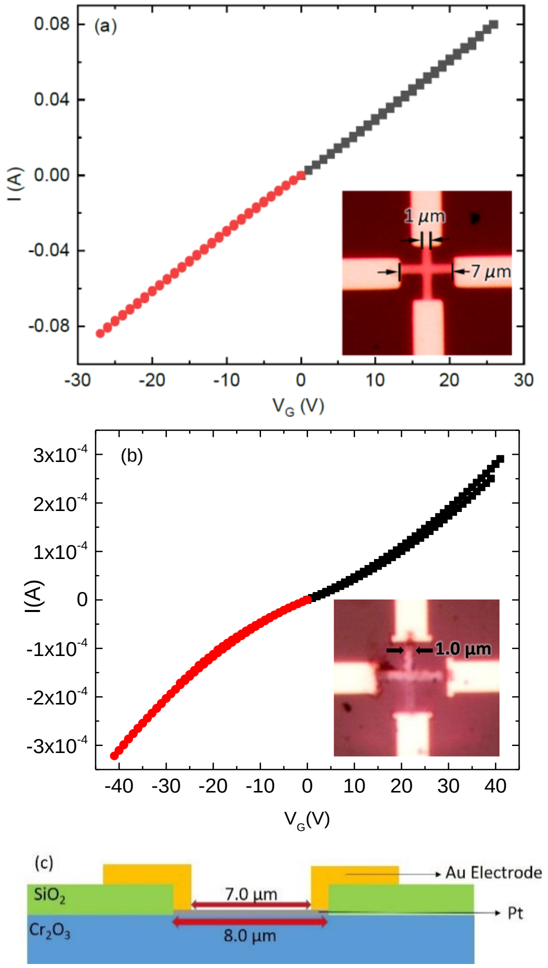

Fig. S3: *I-V* curves. Data are taken at T=300 K for Pt devices based on pure chromia on V_2O_3 (a), and B:Cr_2O_3 on V_2O_3 (b), respectively. The variable gate voltage, V_G is applied across the chromia films of 200 nm thickness using the top Pt Hall bar and the V_2O_3 bottom film as electrodes. The *I-V* curves clearly show the more resistive nature of B-doped film. Also, the non-Ohmic *I-V* characteristic which is more prominent in B:Cr_2O_3 resembles the semiconducting nature of the film. The insets show optical images (top views) of the two Hall bar structures with a size of 1.0 μm × 7.0 μm. The *I-V* data together with the Hall bar geometries allows to estimate the leakage current densities $j = I/A$ flowing through the chromia films with A being the area of the respective Hall cross. (c) shows the cross-section of a schematic of the lithographically fabricated Hall bar structure.

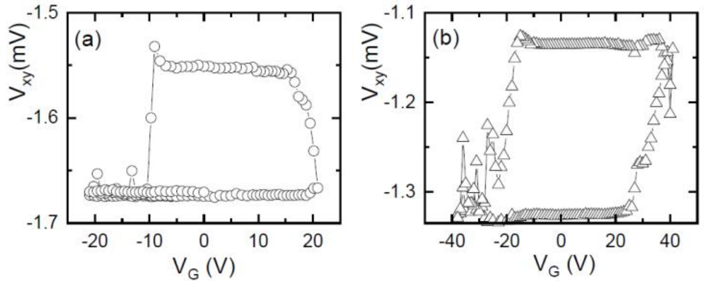

Fig. S4: **High temperature electric hysteresis of Hall-like signal.** (a) V_{xy} versus V_G (circles) measured at T=330 K and $\mu_0 H = -0.85$ T. (b) V_{xy} versus V_G (triangles) of device 2 measured at T = 400 K and $H = 0$.

As mentioned in the main text, Fig S4 shows the hysteresis loops at higher temperatures, indicative of robust nature of toggling of AFM states. (a) shows the switching between two states at 330 K and $\mu_0 H$= -0.85 T, whereas (b) shows the deterministic switching to persist at T =400 K without any magnetic field applied.

In order to show unambiguously that the different states of virtually zero ($V_{xy}^{zero} \approx 0$) and non-zero V_{xy} are magnetically distinct states of B-doped Cr_2O_3 we measured the magnetic field dependence of V_{xy}^{zero} and $V_{xy}^{non-zero}$. Fig. S5 shows the magnetic response of V_{xy}^{zero} (red circles) and $V_{xy}^{non-zero}$ (black squares) at T=300 K measured in a B:Cr_2O_3/Pt Hall bar device. The two different states are initialized by voltage pulses of +24 V (selecting V_{xy}^{zero}) and -25 V (selecting $V_{xy}^{non-zero}$). $V_{xy}^{zero}\ vs. H$ clearly shows a positive slope while $V_{xy}^{non-zero}\ vs. H$ is qualitatively distinct through the virtual absence of magnetic field dependence. This finding strongly supports the magnetically distinct behavior of the two states. The magnetic field dependence of the transverse Hall signal is consistent with spin Hall magnetoresistance. The V_{xy}^{zero} state is associated with in-plane orientation of the Néel vector and thus in-plane orientation of the boundary magnetization. As a result, the applied magnetic field normal to the surface creates maximum torque on the boundary magnetization tilting it out of the plane with increasing applied magnetic field. The increase of the normal component of the boundary magnetization increases the spin Hall magnetoresistance. The $V_{xy}^{non-zero}$ state is characterized by a large spin Hall magnetoresistance already at H=0 consistent with an out-of-plane orientation of the boundary magnetization. In this state, the applied magnetic field and the boundary magnetization are collinear, giving rise to minimal torque on the boundary magnetization, resulting in virtually negligible magnetic field response.

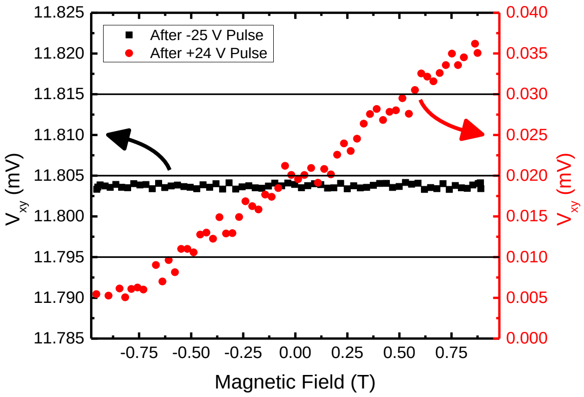

Fig. S5: Magnetic field dependence of the transverse Hall voltage. The two states V_{xy}^{zero} and $V_{xy}^{non-zero}$ are measured in a B:Cr_2O_3/Pt Hall bar device. The two distinct states are prepared by voltage pulses of +24 V (red circles) and -25 V (black squares).

Supplementary Note 4: Kelvin Probe Force Microscopy (KPFM)

To rule out that the MFM contrast variations in Fig. 4 originate from the electrostatic long-range forces, we performed additional Kelvin probe microscopy measurements. Figure S6 shows the topographic image of the entire Hall cross-bar along with its KPFM images acquired 20 minutes after application of +10 V and -10 V voltage pulses of 2 s duration. The miniscule difference in the cross-sectional profiles taken from these KPFM images strongly suggests no contribution of the electrostatic signal to the MFM contrast variations displayed in Fig. 4 in the main text, which, therefore, could be reliably attributed to the magnetization effect.

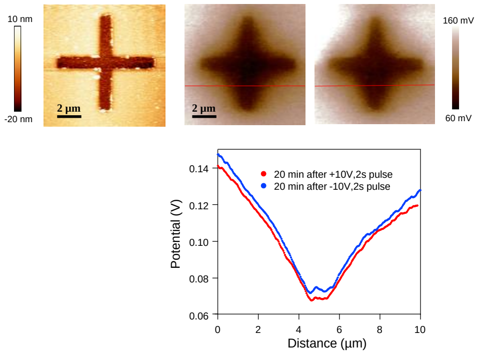

Fig. S6: **Characterization by Kelvin Probe Force Microscopy**. Topographic (left) and KPFM (middle and right) images of the entire Hall cross-bar structure. The plot at the bottom shows the cross-sectional profiles of two KPFM signals taken along the horizontal red lines.

Supplementary Note 5. Topography and two-dimensional conductivity measurements

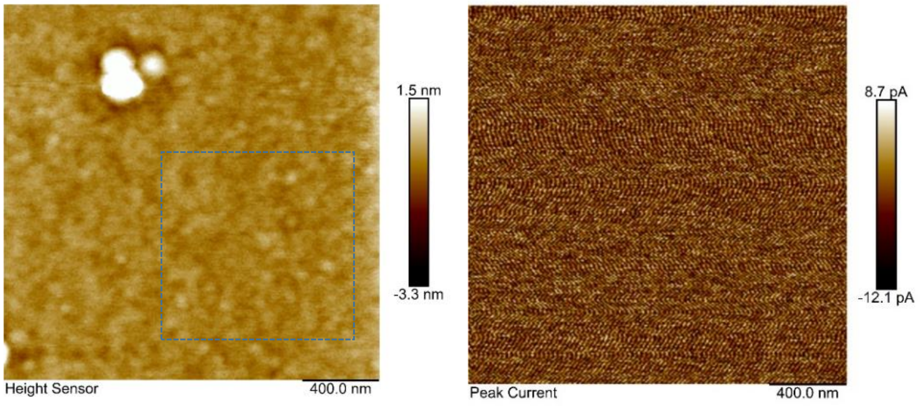

Fig. S7: **Topography and 2D conductivity measurements.** (left) 2 x 2μm AFM image showing the surface topography of the PLD grown B:Cr_2O_3 Chromia film which is the common constituent film for both Hall devices. The box of 1 x 1 μm size represents the area used to analyze the RMS surface roughness of 0.17 nm. (right): Corresponding C-AFM image simultaneously recorded with a DC bias of +0.5 V applied between the tip and back gate V_2O_3 film. At the applied voltage and within tip-surface interaction conditions (30 nN force) there are no leakage pathways associated with defects.

Supplementary Note 6. PFM spectroscopy on pure Chromia

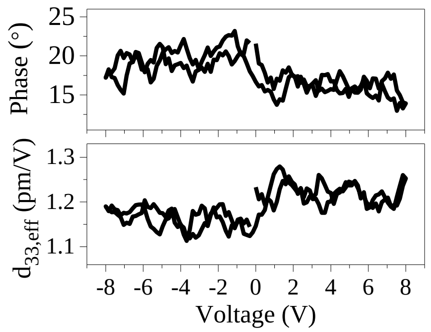

Fig. S8. Local PFM spectroscopy loops. Data obtained through top electrodes in pure Chromia. PFM amplitude (bottom) and phase (top) shows the absence of any induced polarization in pure Chromia. The measurements were done on similar devices as that of B:Cr_2O_3 shown in the main text.

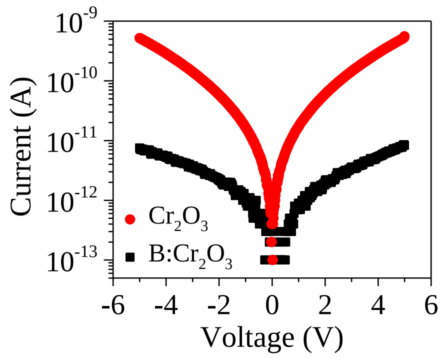

Fig. S9. Comparison of *I-V* curves in pure and B-doped chromia. Data obtained from the capacitive structures fabricated for PFM studies (main text, methods section). It can be seen that the latter is much more resistive than the former. This agrees well with the inverse photoemission data shown in Figure S2.

Supplementary Note 7. Estimation of the effective $d_{33,\mathrm{eff}}$ coefficient

The PFM amplitude signal, A, can be estimated as $A = d_{33,\mathrm{eff}} V Q$, where $d_{33,\mathrm{eff}}$ is the effective piezoelectric coefficient, V is the driving voltage and Q is the quality factor associated with the cantilever dynamics. An order of magnitude estimate of the $d_{33,\mathrm{eff}}$ coefficient can be obtained by comparing the PFM amplitude signal of the material under investigation with the electromechanical response of a reference material with the well-known piezoelectric coefficients measured by independent methods. In our studies, lithium niobate (LNO) thin film crystals were used as the reference sample. PFM testing of LNO and $B{:}Cr_2O_3$ has been carried out using the same cantilevers. The $d_{33,\mathrm{eff}}$ in LNO has been reported to be 8.4 pm/V [4] and by comparing the PFM amplitude signal in LNO [5], a calibration factor for similar cantilevers has been obtained and then used to estimate the $d_{33,\mathrm{eff}}$ value in $B{:}Cr_2O_3$.

Supplementary Note 8. Magnetic anisotropy free energy

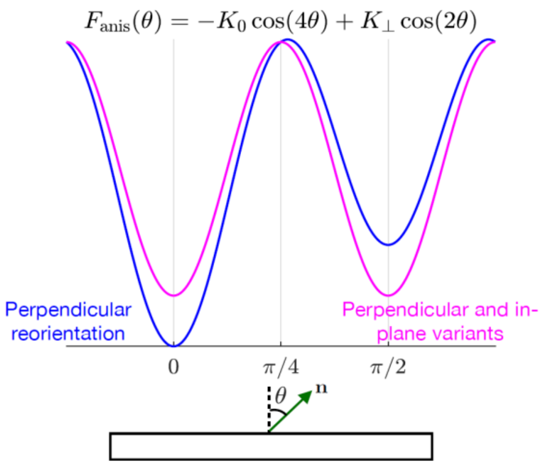

Fig S10. Magnetic anisotropy free energy of the Néel order. The perpendicular anisotropy coefficient $K_\perp$ is altered by the uniform strain produced when Polar Nano Regions (PNRs) align with electric field. For thermally activated switching process, positive value of $K_\perp$ promotes perpendicular orientation, while zero value makes perpendicular and in-plane orientations equally probable.

Supplementary Note 9. Growth and Fabrication Methods

9.1 Pulsed Laser Deposition of Sesquioxides V_2O_3 and B-doped Cr_2O_3

The V_2O_3 films were grown in vacuum by pulsed laser deposition. The Al_2O_3 substrate was cleaned according to a modified RCA protocol which was developed specifically to achieve atomically clean Al_2O_3 substrates [6]. The clean Al_2O_3 substrate was then placed in the growth chamber and the temperature is raised to 750 °C. The system was equipped with a 248 nm KrF excimer laser which is focused on a 2-inch diameter target of V_2O_3 (99.9%). For deposition, the laser was set to operate at 150 mJ energy per pulse and at a repeat rate of 6 Hz. The laser ablates the rotating target (30 Hz) creating a plume of material which is subsequently deposited on the target. The laser is rastered across the rotating target for even wear of the target. The sample itself rotates at 3 Hz to ensure even deposition. The deposition rate is determined by growing several samples of various thicknesses and subsequently measuring the thickness via X-ray reflectivity. After the deposition of the V_2O_3, the doped chromia is grown on top.

The growth of the B-doped chromia is accomplished through the same procedure as above with the following exceptions; a chromia target (99.8%) is used, the temperature of the sample is reduced to 700 °C, the repetition rate of the laser is set to 10 Hz, and the deposition is done in the presence of a partial pressure of decaborane ($B_{10}H_{14}$) gas. Decaborane is solid at room temperature, but highly volatile. A decaborane partial pressure was accomplished by storing a small amount of decaborane in a canister attached to the vacuum chamber with a precision leak valve in between. The decaborane canister was evacuated beforehand (to remove volatile impurities) and then heated to 53 °C prior to deposition, creating a decaborane vapor. The temperature of the decaborane was maintained throughout the entire deposition. The precision leak valve is then used to control the decaborane pressure in the vacuum chamber. The base pressure of the vacuum chamber was 9.1×10^{-8} Torr, while during chromia deposition the pressure was maintained between 1.7×10^{-6} Torr and 1.9×10^{-6} Torr by adjusting the leak valve.

9.2 Magnetron sputtering of Pt layer and lithography of Hall bar devices

A 5 nm thick Pt film was deposited on Cr_2O_3 film via DC magnetron sputtering in a vacuum chamber with base pressure of 1×10^{-8} Torr (1.33×10^{-8} mbar) and a process pressure of

5 mTorr (6.67×10^{-3}mbar) with an applied power of 30W. The heterostructure thus consists of a Pt thin film on top of 200 nm B-doped Chromia film grown on a back gate V_2O_3 film (20 nm). A schematic of the heterostructure is shown in Fig. S3 (c). A two-step E-beam lithography procedure was used to fabricate the Hall bar devices. First, a negative tone resist was patterned in the form of Hall bars of sizes 1.5 x 8 μm and 1.0 x 8 μm and subsequently developed. Ar ion etching was used to etch away the exposed Pt layer while protecting the Hall bars. Next, a 30 nm SiO_2 dielectric spacer layer was deposited via RF sputtering onto the patterned sample, followed by lift-off. In the second lithography step, a positive tone resist was patterned to fabricate wires and contact pads. Finally, Ti/Au (3 nm/50 nm) electrodes were deposited using e-beam assisted evaporation. This step was followed by final lift-off in acetone to reveal the finished devices. It is important to note that the leakage profile of 30 nm SiO_2 film was individually determined by *I-V* measurements on films deposited on sapphire substrates. Upon applying 10 V on the top and bottom contact pads across a 30 nm SiO_2 film, a current of 20 nA was measured, thus denoting its ability to withstand sizeable voltage levels.

9.3 Molecular beam Epitaxy of CoPt multilayers with perpendicular and in-plane anisotropy

The CoPd films were grown using molecular beam epitaxy. The thin film ferromagnet was grown under ultra-high vacuum, with a background pressure of ~5×10^{-9} mbar during growth. After introduction to the vacuum the sample was heated to 300 °C. The growth rate of the Co and Pd has been previously gauged by growing a series of films of various thicknesses and characterizing the thicknesses by X-ray reflectivity. The resulting linear growth rate is extrapolated down for films of single nanometer proportions. In addition, the growth rate was monitored in-situ, by a quartz crystal thin-film thickness monitor which has been previously calibrated. Both methods provided the same measurement for film thickness.

10 Supplementary Characterization Methods

10.1 Vibrating Sample Magnetometry

The Quantum Design VersaLab vibrating sample magnetometer is designed for magnetic characterization over a large temperature range. When placed in the sample holder, the temperature of the sample can be controlled from 50 K to 400 K. The vibrating sample magnetometer operates by moving a magnetic sample through stationary pickup coils. By measuring the induced current in the coils the moment of the magnetic sample can be calculated. Using the integrated lock-in measurement technique the magnetometer has a noise floor of $< 6.0 \times 10^{-7}$emu. For the measurements shown in Figure 2, the temperature of the sample was set between 66 K and 400 K in intervals of 4 K. The sample temperature was allowed to stabilize before the measurement was made. By sweeping the magnetic field, the hysteresis loop of the sample was measured. The coercive field values were taken as the field values at which the measured magnetization crossed zero, the exchange bias was then calculated as the average of the two coercive field values.

10.2 Polar Kerr Magnetometry

The data shown in Fig. 1 of the main text were taking using polar Kerr magnetometry. The sample is placed on a sample holder inside a temperature-controlled cryostat with quartz optical windows. The sample holder is then placed inside GMW electromagnet with a 4 mm axial hole drilled though one of the poles of the electromagnet to accommodate the laser light. A 532 nm wavelength 5 mW laser diode was used and polarized using a Glans-Thomson polarizer. The light was then directed to be incident on the normal of sample, through the axial hole of the electromagnet. By reflecting the light off the sample back through the axial hole of the electromagnet, it is ensured that the polar alignment of both the laser and the applied magnetic field is excellent. By means of a beamsplitter, the reflected light is directed through a photoelectric modulator, a final polarizer, and finally a photodiode. The photoelectric modulator, uses a 50 kHz electrical signal to modulate the laser light beam. The optical axis of the modulator is aligned parallel to the initial Glans-Thomson polarizer. The same 50 kHz signal which modulates the laser is fed into a lock-in amplifier as a reference signal. The final polarizer is oriented at 45° with respect to the initial polarizer and the photoelectric modulator. Any deviation of the polarization plane

of the light due to the interaction of the light and magnetization of the sample through the magneto-optical Kerr effect will affect the transmission through the second polarizer. These differences were detected by a photodiode which is used as an input to the lock-in amplifier. An in-depth description of the Kerr magnetometry used here can be found in Ref [7]. By cycling the magnetic field, a hysteresis loop can be observed in the signal, corresponding to the magnetization of the sample. A linear background is also present corresponding to Faraday rotation of the light as it passes through the quartz window in the presence of a magnetic field. The linear background was subtracted and the hysteresis loops are normalized between 1 and -1. After normalization, the coercive field values of the hysteresis loop are taken to be the zeroes of the data.

10.3 Spin Resolved Inverse Photoemission Spectroscopy

The spin-polarized inverse photoemission (SPIPES) spectra were taken at various different temperature. The magnetic and electric field were both applied to achieve field cooling from 500 K to 300 K with $B \cdot E < 0$. After field cooling, the spectra were taken at ~300 K and sample was gradually heated to targeted temperature (~315 K, ~325 K) and corresponding spectra were taken when the thermal equilibrium was achieved. Our spin-polarized inverse photoemission experiments utilize a transversely polarized spin electron gun based upon the Ciccacci design [8, 9]. As described elsewhere [9], the spin electron gun was used in combination with an iodine-based Geiger-Müller isochromat photon detector with a SrF_2 window. As is typical of such instruments, the electron gun has 28% spin polarization, and the data have been corrected for this incident gun polarization. The direction of electron polarization is in the plane of the sample. The magnetoelectric cooling was accomplished in an axial magnetic field of > 40mT and a voltage of 3 kV applied across the film thickness. The Fermi level was established from tantalum and gold foils in electrical contact with the sample. We find that the in-plane spin polarization increases with increasing temperature over the range 300 to 325 K in spin-polarized inverse. Since the spin asymmetry is increasing in a region where the boundary magnetization is decreasing, this generally indicates that the in-plane polarization increases faster than the boundary magnetization declines. Independent validation of the spin-polarized inverse photoemission, measuring the in-plane polarization Fig. S9 (c), comes from in-plane X-ray

magnetic circular dichroism Fig. S9 (a), which provides a small, but nonetheless non-zero signal at the Cr L3 ($2p_{3/2}$) edge indicative of a small in-plane Cr^{3+} moment Fig S9 (b) at 320 K. X-ray absorption (XAS) and X-ray magnetic circular dichroism (XMCD) studies were carried out at Canadian Light Source. Spatial resolution of the Elmitec GmbH PEEM microscope is better than 30 nm for an ideal flat sample. The spectra were taken in thermal equilibrium conditions estimated about ~320 K based on the reading of a thermocouple. Both "up" and "down" domains were observed in XMCD and the spectra closely resemble those taken of chromia and reported elsewhere [10]. Note that the XMCD spectra showing net boundary moments and the SPIPES spectra with the largest spin asymmetry were taken in the temperature region of ~320 K which is higher than the Néel temperature of pure Cr_2O_3 indicating an enhanced magnetic ordering temperature with boron doping.

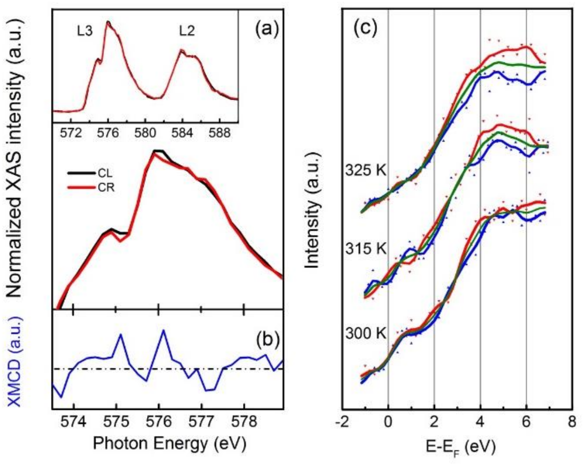

Fig. S11 X-ray absorption spectra, X-ray magnetic circular dichroism, and spin-polarized inverse photoemission spectra of B: Cr_2O_3. (a) XAS spectra at Cr L_3 edges for B-doped Cr_2O_3 thin film. The inset shows the wider XAS energy range across both L_2 and L_3 edges. The spectra were taken at ~320 K. The polarity of light was label as CL (black) and CR (red) for circular left and right polarization respectively. (b) XMCD spectrum at Cr L_3 edges. (c) Spin-polarized inverse photoemission spectra taken at different temperature as labeled after field cooling with $B \cdot E < 0$. Spin majority-spin up state and spin minority-spin down state are indicated by blue and red markers respectively. The green lines are spin integrated spectra.

10.4 Hall measurements

Prior to taking Hall measurements, the electrical resistance of Pt films of various thicknesses deposited on Al_2O_3 substrates was determined. The resistivity of 5 nm Pt film

was determined to be 9.87 x 10^{-7} Ω-m by two probe measurements on wires of length 1.6 mm and width 100 µm deposited through a shadow mask. The Hall measurements are taken by sourcing the current through a Keithley sourcemeter 6221 and measuring the transverse voltage via Keithley K2182 Nanovoltmeter. This setup allows for a Delta measurement where the current directions are reversed and the voltage signal is measured for both directions. This methodology helps in reducing systematic errors by cancelling out the voltage contributions from thermoelectric EMFs, a common source of noise in low voltage measurements. The electric field across the heterostructure was provided by applying voltage across the back gate V_2O_3 film and one of the Hall bar contact pads.

10.5 Scanning Probe Microscopy

Scanning probe microscopy was performed to characterize the morphology and leakage properties of the heterostructure. As shown in the supplementary Fig S4. (a) a 2 x 2 µm Atomic Force Microscopy (AFM) scan of the Cr_2O_3 surface is recorded. The root mean square (RMS) roughness of a 1 x 1 µm area (shown with a box) on the film is determined to be 0.17 nm. Together the RMS value and the peak to peak height difference ensure that the Pt film deposited on top is continuous and without any voids. An AFM conductivity mapping (C-AFM) recorded simultaneously with topography image is also shown (supplementary Fig 3 (b)), where the image is taken with DC bias of +0.5V maintained between the bottom V_2O_3 film and the metallic tip. The instrument used for these measurements is the Bruker AFM in PeakForce Tunneling AFM (TUNA) in the tapping mode. In this mode of operation, the tip is intermittently made to contact the sample surface, thus avoiding the lateral forces during imaging. The conductivity image shows no leakage through the heterostructure, a condition essential to maintain sizeable electric field across the device. Since the AFM tip is never in ohmic contact with the surface, the situation is different than actual devices where the Pt hall bars are in contact with a larger area on the Cr_2O_3 film and hence a minimal leakage is present. Nevertheless, the C-AFM is an excellent technique in determining any structural defects on the surface that are

responsible for enhanced leakage rendering the device unsuitable for application of essential electric field, as has been reported in our earlier study [11].

Magnetic force microscopy (MFM) utilizes the long-range force, originating from interaction between a magnetized tip and the gradient of the magnetic stray field of the sample surface. By maintaining a tip lift-height of 30 nm the van der Waals interaction becomes negligible while the magnetic signal dominates. ASYMMFMHM tips with high magnetic moment CoCr coating were used for MFM imaging.

Kelvin probe Force Microscopy (KPFM) is another technique of the electrical characterization methods available in the ambit of scanning probe microscopy. KPFM provides information about the surface potential or work function of a sample surface. For this work surface potential of the Hall bar devices after applying voltages with opposite polarities was acquired. Metallic tips with HQ:NSC 18/Pt cantilevers from MikroMasch were used for KPFM imaging. It is pertinent to mention that both MFM and KPFM imaging was acquired using the commercial AFM system (MFP-3D, Asylum Research).

Supplementary References

[1] W. Echtenkamp, M. Street, A. Mahmood, C. Binek, *Tuning the Effective Anisotropy in a Voltage-Susceptible Exchange-Bias Heterosystem*, Physical Review Applied, **7** (2017) 034015.
[2] C.-P. Kwan, R. Chen, U. Singisetti, J.P. Bird, *Electric-field dependent conduction mechanisms in crystalline chromia*, Applied Physics Letters, **106** (2015) 112901.
[3] N. Wu, X. He, A.L. Wysocki, U. Lanke, T. Komesu, K.D. Belashchenko, C. Binek, P.A. Dowben, *Imaging and Control of Surface Magnetization Domains in a Magnetoelectric Antiferromagnet*, Physical Review Letters, **106** (2011) 087202.
[4] A. Labuda, R. Proksch, *Quantitative measurements of electromechanical response with a combined optical beam and interferometric atomic force microscope*, Applied Physics Letters, **106** (2015) 253103.
[5] H. Lu, Y. Tan, J.P.V. McConville, Z. Ahmadi, B. Wang, M. Conroy, K. Moore, U. Bangert, J.E. Shield, L.-Q. Chen, J.M. Gregg, A. Gruverman, *Electrical Tunability of Domain Wall Conductivity in $LiNbO_3$ Thin Films*, Advanced Materials, **31** (2019) 1902890.
[6] D. Zhang, Y. Wang, Y. Gan, *Characterization of critically cleaned sapphire single-crystal substrates by atomic force microscopy, XPS and contact angle measurements*, Applied Surface Science, **274** (2013) 405-417.
[7] S. Polisetty, J. Scheffler, S. Sahoo, Y. Wang, T. Mukherjee, X. He, C. Binek, *Optimization of magneto-optical Kerr setup: Analyzing experimental assemblies using Jones matrix formalism*, Review of Scientific Instruments, **79** (2008) 055107.

[8] M. Street, W. Echtenkamp, T. Komesu, S. Cao, P.A. Dowben, C. Binek, *Increasing the Néel temperature of magnetoelectric chromia for voltage-controlled spintronics*, Applied Physics Letters, **104** (2014) 222402.
[9] K. Takashi, W. Carlo, J. Hae-kyung, P.P. David, T. Rammer, E.J. Marty, J.G. Tim, A.D. Peter, *Apparatus for spin-polarized inverse photoemission and spin scattering*, in: Proc. SPIE, 2000.
[10] K. Toyoki, Y. Shiratsuchi, T. Nakamura, C. Mitsumata, S. Harimoto, Y. Takechi, T. Nishimura, H. Nomura, R. Nakatani, *Equilibrium surface magnetization of α-Cr_2O_3 studied through interfacial chromium magnetization in Co/α-Cr_2O_3 layered structures*, Applied Physics Express, **7** (2014) 114201.
[11] A. Mahmood, M. Street, W. Echtenkamp, C.P. Kwan, J.P. Bird, C. Binek, *Dielectric properties of thin Cr_2O_3 films grown on elemental and oxide metallic substrates*, Physical Review Materials, **2** (2018) 044401.
